# Effect of *CCR5-*Δ*32* Heterozygosity on HIV-1 Susceptibility: A Meta-Analysis

**DOI:** 10.1371/journal.pone.0035020

**Published:** 2012-04-04

**Authors:** SiJie Liu, ChuiJin Kong, Jie Wu, Hao Ying, HuanZhang Zhu

**Affiliations:** The State Key Laboratory of Genetic Engineering, Institute of Genetics, School of Life Science, Fudan University, Shanghai, China; National Institute of Health, United States of America

## Abstract

**Background:**

So far, many studies have investigated the distribution of CCR5 genotype between HIV-1 infected patients and uninfected people. However, no definite results have been put forward about whether heterozygosity for a 32-basepair deletion in CCR5 gene (CCR5-Δ32) can affect HIV-1 susceptibility.

**Methods:**

We performed a meta-analysis of 18 studies including more than 12000 subjects for whom the CCR5-Δ32 polymorphism was genotyped. Odds ratio (OR) with 95% confidence interval (CI) were employed to assess the association of CCR5-Δ32 polymorphism with HIV-1 susceptibility.

**Results:**

Compared with the wild-type CCR5 homozygotes, the pooled OR for CCR5-Δ32 heterozygotes was 1.02 (95%CI, 0.88–1.19) for healthy controls (HC) and 0.95 (95%CI, 0.71–1.26) for exposed uninfected (EU) controls. Similar results were found in stratified analysis by ethnicity, sample size and method of CCR5-Δ32 genotyping.

**Conclusions:**

The meta-analysis indicated that HIV-1 susceptibility is not significantly affected by heterozygosity for CCR5-Δ32.

## Introduction

Inter-individual variability in susceptibility to HIV-1 infection, transmission, disease progression, and response to antiviral therapy has been attributed to host variability in multiple genes [1]. CC chemokine receptor 5 (CCR5) and CXC chemokine receptor 4 (CXCR4) are co-receptors for the entry of human immunodeficiency virus type 1 (HIV-1) into target cells [2]. A natural knockout deletion of 32 bases in CCR5 gene introduces a premature stop codon resulting in truncated protein product [3]. People homozygous for CCR5-Δ32 are naturally resistant to R5 HIV infection and the heterozygous state is associated with up to 2–4 years delay in disease progression [4] ^,^. Recently, Allers et al reported that they have successfully cured a HIV infected patient through CCR5-Δ32/Δ32 stem cell transplantation [5], [6]. On the other hand, the evidence for protection from HIV-1 infection among CCR5-Δ32 heterozygotes is mixed. A meta-analysis of Despina et al suggested that perinatal infection rates are not strongly determined by the number of functional CCR5 receptors in the children [7]. For adults, some studies have reported that CCR5-Δ32 heterozygotes could be protective against HIV transmission [8]–[15], whereas others have not confirmed that [16]–[28]. Therefore, we performed a meta-analysis of the accumulated data to address this question definitively.

## Materials and Methods

### Search Strategy and Study Selection

English database of Google Scholar (GS), PubMed and Chinese database of CNKI were searched till June 2011 using key words: CCR5-Δ32 and HIV-1. Studies satisfying the following criteria were included: case-control studies reporting the association of CCR5-Δ32 genotype with HIV-1 susceptibility, distribution of CCR5-Δ32 genotype between the cohorts was shown, not a prenatal HIV-1 infection study.

### Data Extraction and Statistical Analysis

Two reviewers( SiJie Liu, Jie Wu) independently performed data extraction and then checked the results together. The following information was extracted from included studies: authors, year of publication, ethnicity, country, sample size, method of CCR5-Δ32 genotyping and CCR5-Δ32 genotype of cohorts.

Odds ratio (OR) and its 95% confidence intervals (CI) were used to evaluate the association of CCR5-Δ32 heterozygotes with HIV-1 susceptibility. Subgroups were identified by ethnicity, sample size and method of CCR5-Δ32 genotyping. A chi-square-based Q-test was carried out to assess heterogeneity across studies [29]. A *P* value less than 0.10 was used to denote statistical significance. Fixed effects (Mantel and Haenszel) model was employed to pool the effects of studies without heterogeneity, otherwise the random effects (Dersirmonian and Laird) model was used [30], [31]. Publication bias was evaluated by Egger’s and Begg’s test with funnel plots [32], [33]. Asymmetry of the funnel plot suggests publication bias. A *P* value less than 0.05 was used to denote statistical significance. One-way sensitivity analyses were performed to examine the influence of individual studies on meta-analysis’s results. Data were analyzed using Stata version 10.0 (StataCorp, College Station, Tex).

## Results

**Figure 1:**
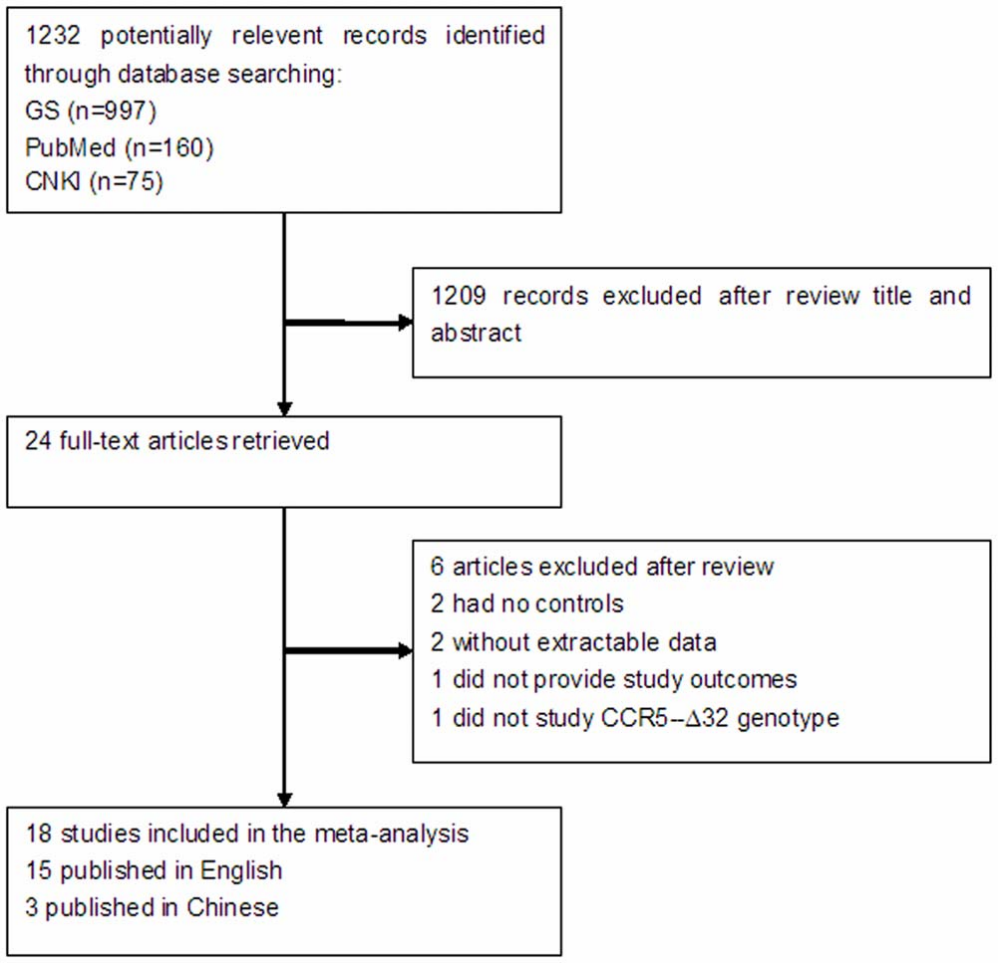
Selection process of studies included in the meta-analysis.

Figure 1 summarized the selection process of literatures. The electronic search yielded 1232 records, after screening over titles and/or abstracts, 24 articles were selected for further review. Finally, 18 studies involving 6427 cases and 5809 controls were included in the meta-analysis. Study sample size ranged from 140 to 2605 subjects. Study characteristics of the 18 eligible studies were summarized in Table 1. Distribution of CCR5 genotype among subjects was shown in Table 2. Briefly, 9 studies involved Caucasian subjects [8], [10]–[12], [17], [22]–[24], 4 studies involved Mongoloid subjects [16], [21], [25], [28], 3 studies involved African subjects [8], [12], [24], 3 studies involved Latina subjects [12], [18], [27]. In addition to CCR5-Δ32 genotype, 8 studies provided the subjects’ CCR2-64I genotype [10], [16], [19]–[21], [26]–[28], 5 studies provided the subjects’ SDF-1 genotype [16], [20], [21], [26], [27]. All studies were done in subjects of mixed genders except that by Downer et al [24], which only included women.

**Table 1:** Characteristics of selected studies in the meta-analysis.

Study (year)	Country	Genotyping method	Ethnicity	Sample size (case/control)
Battiloro (2000) [17]	Italy	PCR	Caucasian	256/806
Deng (2004) [28]	China	PCR	Mongoloid	88/119
Diaz (2000) [18]	Colombia	PCR	Latina	29/188
Downer (2002) [24]	USA	PCR-RFLP	Mixed	929/445
Grimaldi(2002)[13]	Brazil	PCR	Mixed	113/549
Li (2003) [26]	China	PCR	Mongoloid	94/46
Liu (2004) [20]	USA	PCR	Mixed	316/513
Lockett (1999) [19]	Britain	PCR	Mixed	86/105
Oh(2008)[8]	German	PCR	Caucasian	610/427
			African	35/26
Papa (2000) [10]	Greece	PCR	Caucasian	138/239
Paz-y-Mino (2005) [27]	Ecuador	PCR	Latina	295/50
Philpott (2003) [12]	USA	PCR-RFLP	Mixed	2047/558
Takacova (2008) [22]	Slovakia	PCR	Caucasian	162/198
Tan (2010) [16]	China	PCR	Mongoloid	250/237
Tang (2010) [25]	China	PCR-LDR	Mongoloid	245/223
Trecarichi(2006)[11]	Italy	PCR	Caucasian	120/120
Veloso(2010)[23]	Span	PCR	Caucasian	184/236
Wang (2003) [21]	China	PCR	Mongoloid	330/474

**Table 2:** Distribution of CCR5 genotype of included studies.

Study	Ethnicity	HIV-infected		Healthy Controls		Exposed but uninfected	
		+/+^1^	+/△^2^	+/+	+/△	+/+	+/△
Battiloro	Caucasian	232	24	744	62		
Deng	Mongoloid	88	0	117	2		
Diaz	Latina	28	1	142	8	37	1
Downer	Mixed	879	50	422	23		
Grimaldi	Mixed	103	10	520	29		
Li	Mongoloid	90	4			45	1
Liu	Mixed	261	55	354	68	69	22
Lockett	Mixed	63	23	38	10	40	17
Oh	Caucasian	595	115	352	75		
	African	35	0	25	1		
Papa	Caucasian	132	6	216	23		
Paz-y-Mino	Latina	292	3	50	0		
Philpott	Mixed	1940	107	513	45		
Takacova	Caucasian	137	25	164	34		
Tan	Mongoloid	226	24			222	15
Tang	Mongoloid	221	24			209	14
Trecarichi	Caucasian	111	9			24	6
Veloso	Caucasian	144	40	174	26	31	5
Wang	Mongoloid	329	1			473	1

1 CCR5 homozygotes.

2 CCR5-Δ32 heterozygotes.

**Figure 2:**
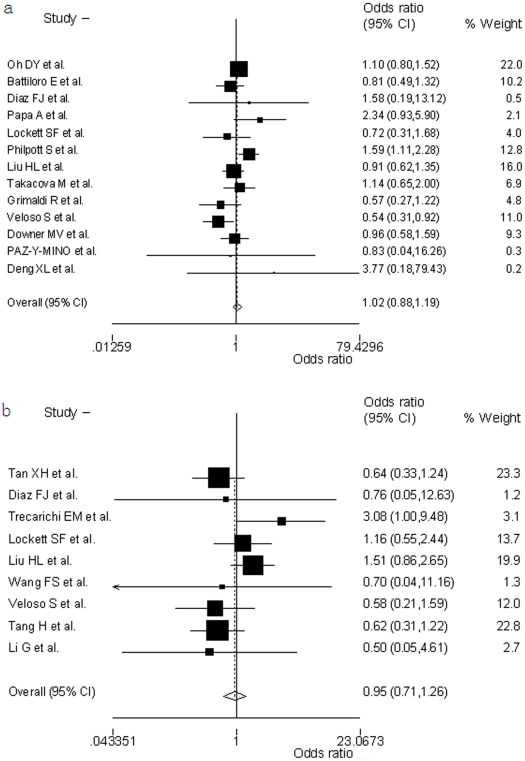
Odds ratio of HIV-1 infection of CCR5-Δ32 heterozygotes versus wild type CCR5 homozygotes. The area of the black square reflects the weight of each study. The diamonds represent the combined odds ratio and 95% confidence interval using the fixed effects model for (a) healthy controls (HC) and (b) exposed uninfected controls (EU).

**Figure 3:**
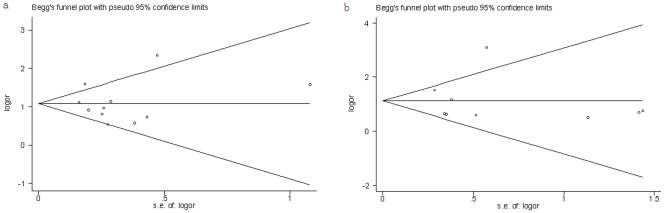
Funnel plots to detect publication bias in the meta-analysis. (a) Healthy controls considered; (b) exposed uninfected controls considered. The horizontal line indicates the pooled log odds ratio (OR) and guidelines to assist in visualizing the funnel are pooled at 95% pseudo confidence limits for this estimate.

Compared with the wild-type CCR5 homozygotes, the pooled OR for CCR5-Δ32 heterozygotes was 1.02 (95%CI, 0.88–1.19, *p* = 0.073) for healthy controls (HC) (figure 2a) and 0.95 (95%CI, 0.71–1.26, *p* = 0.182) for exposed uninfected (EU) controls (figure 2b). There was no significant between-study heterogeneity. No asymmetry is observed in the funnel plots (figure 3a, 3b).

**Table 3:** Stratified analysis of CCR5-Δ32 heterozygotes and susceptibility to HIV-1.

Variable	Healthy Controls		Exposed but uninfected	
	OR (95%CI)	*P* 1	OR (95%CI)	*P*
Ethnicity				
African	1.17 (0.71–1.93)	0.604		
Caucasian	1.06 (0.73–1.53)	0.005	1.31 (0.25–6.85)	0.029
Latina	1.28 (0.62–2.61)	0.942		
Mongoloid			0.63 (0.39–1.01)	0.995
Genotyping Method				
PCR	0.94 (0.79–1.12)	0.239	1.04 (0.76–1.44)	0.233
PCR-RFLP	1.32 (0.99–1.78)	0.110		
Sample Size				
>800	1.09 (0.91–1.30)	0.152		
<800	0.87 (0.65–1.16)	0.144	0.81 (0.57–1.14)	0.233

1 *P* value of Q-test for heterogeneity test.

We also performed stratified analysis by ethnicity, sample size and method of CCR5-Δ32 genotyping. The results were summarized in Table 3. All the results were consisted with overall analysis and no publication bias were observed.

Sensitive analysis was conducted by deleting one study at a time to examine the influence of individual data-set to the pooled ORs. All of the corresponding pooled ORs were not materially altered (Data not shown).

## Discussion

Meta-analysis offers a powerful method to synthesize information of independent studies with similar intention. It has been proved that CCR5-Δ32 homozygotes are associated with near complete protection to HIV-1 infection. Moreover, published data have demonstrated that a disease-retarding effect of CCR5-Δ32 heterozygosity in HIV-1 infected individuals [34]. Whereas it remained unclear if a heterozygosity for CCR5-Δ32 could affect HIV-1 susceptibility. Thus we performed this meta-analysis involving 18 eligible studies with 6427 patients and 5809 controls. The study demonstrated that CCR5-Δ32 heterozygosity has little or no protective effect against HIV-1 infection among adults. This result is similar to a previous study of perinatal HIV-1 infection [7]. Several factors might underlie the lack of observed association between CCR5-Δ32 heterozygosity and HIV-1 susceptibility. First, the expression of CCR5 is influenced by factors other than CCR5 genotype. Even an individual with CCR5-Δ32 heterozygosity could still express high level of CCR5 [35], [36]. Second, susceptibility to HIV-1 infection is affected by a combination of genes besides CCR5. The CCR5-Δ32 heterozygotes couldn’t provide a full resistant to HIV-1 infection as the homozygotes. It is possible that a single Δ32 allele exerts a protective effect against HIV-1 infection only if it occurs combined with other protective factors [9].

There are a number of limitations to our study. First, although test of publication bias have generated negative results, studies solely in conference or in local journals may have been overlooked. Second, HIV-1 of X4 strain take advantage of CXCR4 as co-receptor. It has been reported that new infections in individuals are primarily established by strains that use R5 [37]–[39]. Currently, it is remains controversial about if HIV could use CXCR4 as co-receptor in primary HIV infection [40]. Primary infection with CXCR4-using HIV-1 strains is believed to be a rare event [41]. Thus, we might believe that in most of the cases HIV-1 R5 strain cause the initial infection, rather than the X4 strain. Although we couldn’t exclude the interference of X4 viruses, it is unlikely that virus of X4 strain would significantly affect the results. Third, controls of some studies were solely derived from healthy individuals. For studies concerning disease susceptibility, it’ll be more proper to take samples from exposed uninfected people as controls. Fourth, susceptibility to HIV-1 is influenced by multiple factors other than CCR5, they might interfere the precision of analysis.

In conclusion, our study involving more than 12000 subjects suggested that CCR5-Δ32 heterozygosity has little effect on protecting from HIV-1 infection. Therefore, other chemokine receptors and transmission mechanisms may play a more important role.
